# Methylation of *MTHFR* Moderates the Effect of Smoking on Genomewide Methylation Among Middle Age African Americans

**DOI:** 10.3389/fgene.2018.00622

**Published:** 2018-12-10

**Authors:** Allan M. Andersen, Man-Kit Lei, Robert A. Philibert, Steven R. H. Beach

**Affiliations:** ^1^Department of Psychiatry, University of Iowa, Iowa City, IA, United States; ^2^Department of Sociology, University of Georgia, Athens, GA, United States; ^3^Center for Family Research, University of Georgia, Athens, GA, United States; ^4^Behavioral Diagnostics, Coralville, IA, United States; ^5^Department of Psychology, University of Georgia, Athens, GA, United States

**Keywords:** smoking, epigenetics, DNA methylation, AHRR, cg05575921, GPR15, cg19859270, digital PCR

## Abstract

Differential methylation at *MTHFR* (m*MTHFR)* has been examined previously as a moderator of changes in methylation among nascent smokers, but the effects of mMTHFR on genomewide patterns of methylation among established smokers in middle age are unknown. In the current investigation we examined a sample of 180 African American middle-aged smokers and non-smokers to test for patterns indicative of three different potential mechanisms of impact on epigenetic remodeling in response to long-term smoking. We found that m*MTHFR* moderated the association between smoking and changes in methylation for more than 25% of the 909 loci previously identified as being associated with smoking at a genomewide level of significance in middle-aged African Americans. Observed patterns of effect indicated amplification of both hyper and hypo methylating responses to smoking among those with lower m*MTHFR*. Moderating effects were robust to controls for sex, age, diet, and cell-type variation. Implications for potential mechanisms conferring effects are discussed. Of particular potential practical importance was a strong effect of m*MTHFR* on hypomethylation at GPR15 in response to smoking, indicative of the differential impact of MTHFR activity on changes in a specific cell population linked to inflammatory disease in smokers.

## Introduction

Smoking is the leading cause of preventable morbidity and mortality in the United States, with enormous implications for population-level health (Mokdad et al., 2004; DHHS, 2014). It exerts these effects, for the most part, by increasing liability to complex disorders such as cancer, chronic obstructive pulmonary disease (COPD), type 2 diabetes (T2DM) and obesity, which in turn contribute to early death, chronic illness, and strain on the health care system (Jamal et al., 2018). Better understanding of the moderators of response to smoking at the level of DNA methylation may provide useful clues regarding mechanisms leading to illness, allow assessment of individual risk for developing particular chronic illnesses, and facilitate the identification of high-risk populations. African Americans are of particular interest because they are more likely than European Americans to suffer adverse outcomes for many smoking-related illnesses such as lung cancer and heart disease, even after adjustment for potential confounding covariates such as access to health care (Haiman et al., 2006; Control CfD prevention, 2010). This suggests the value of attention to African American samples in examining factors that may interact with smoking to predict differential methylation.

Changes in methylation due to smoking have been demonstrated at numerous loci across the genome (Andersen et al., 2015), suggesting that extensive epigenetic remodeling occurs in smokers, particularly in long-term smokers. The most consistent association reported for both nascent and long-term smokers has been decreased methylation at the CpG cg05575921, located in the gene for the aryl hydrocarbon receptor repressor (*AHRR*). (Monick et al., 2012; Philibert et al., 2013; Shenker et al., 2013a; Zeilinger et al., 2013; Elliott et al., 2014; Zhang et al., 2014). The effect at *AHRR* appears to be robust across ethnicities (Dogan et al., 2014) as well as age groups (Philibert et al., 2012).

The biochemical basis for sensitivity of methylation at cg05575921 to smoking is interesting and provides potential clues regarding the multiple ways that smoking may affect later health outcomes. When tobacco smoke is inhaled, blood passing through the lungs absorbs large amounts of dioxins and polyaromatic hydrocarbons (PAH) (Talhout et al., 2011), leading to rapid and robust activation of the aryl hydrocarbon receptor (AHR), a key regulator of the xenobiotic response. In turn, activation of AHR leads to activation of CYP1A1, CYP1A2, and CYP1B1, enzymes involved in metabolic breakdown of the toxins. However, if left unregulated, excessive activation of the xenobiotic pathway can result in the production of reactive oxygen species (ROS), catabolism of key cellular components, and potentially carcinogenesis (Ramadoss et al., 2005). Therefore, because of the potential for cellular harm, the xenobiotic pathway is under tight regulatory control, and activation of AHR leads rapidly to activation of an opposing regulatory response. Specifically, following activation of the pathway, a second nuclear receptor, AHRR, is expressed that then reduces the effects of AHR activation by competing for its obligate heterodimer partner, the aryl hydrocarbon nuclear translocator (ARNT), and for DNA binding motifs for AHR. The CpG cg05575921, located in the enhancer region of *AHRR*, has been hypothesized to play a regulatory role in this increase in AHRR availability and subsequent down regulation of the xenobiotic pathway through the recruitment of methylation-specific transcriptional activators (Zeilinger et al., 2013).

In summary, rapid hypo methylation of *AHRR* in response to smoking results from an initial increase in activity of the xenobiotic pathway and AHR, and then secondarily, a robust down regulating response by AHRR to prevent widespread cellular damage from over activation of the pathway. Less clear, however, is the extent to which prolonged activation of the xenobiotic pathway, as compared with other effects of smoke exposure such as direct tissue damage, contributes to epigenetic remodeling observed in smokers, as well as the extent to which differential regulation of the xenobiotic pathway may modify this impact.

Beach and others (Beach et al., 2017) showed that degree of methylation at cg05575921 in response to nascent smokers was moderated by methylation at *MTHFR* (m*MTHFR*). Specifically, they observed greater hypo methylation at cg05575921 in two independent samples (*N* = 293; *N* = 368) of African American young adults among those with greater m*MTHFR*. Given the regulatory role of AHRR over the AHR system, this suggests potential influence over patterns of genomewide change among long-term users as well. In particular, we were interested in three potential patterns that could relate m*MTHFR* to epigenetic remodeling among long-term smokers.

First, as suggested by Beach et al. (2017), because methylene tetrahydrofolate reductase (*MTHFR*) is the rate-limiting enzyme in the methyl cycle, and largely determines the availability of the biologically usable form of folate, 5-methylenetetrahydrofolate, m*MTHFR* could be a source of individual differences in efficiency of methylation repair activities (Stover, 2009), and so account for individual variability in the degree of hypo methylation experienced by smokers. This would be reflected in an interaction of smoking with m*MTHFR* to predict loci that typically are demethylated in response to smoking.

Second, because a more robust response of AHRR limits the potential cellular damage and epigenetic remodeling initiated by AHR activity, it is possible that greater m*MTHFR* will be protective against widespread epigenetic change in response to smoking, and be reflected in both hypo and hyper methylating effects. That is, because greater m*MTHFR* is associated with greater demethylation at cg05575921 in response to smoking, and hence a more robust response of AHRR, it should result in dampened epigenetic remodeling. Conversely, to the extent that AHR activation plays an important protective role in clearing smoke borne toxins systemically, greater m*MTHFR* might lead to amplified epigenetic remodeling for loci that respond to smoking with either hypo or hyper methylation. In this case, because greater m*MTHFR* leads to a reduced activity of AHR, if heightened systemic toxin exposure drives epigenetic remodeling, greater m*MTHFR* and therefore diminished AHR activity would increase the impact of smoking on epigenetic change.

Given the potential for a significant impact of m*MTHFR* status on the epigenetic effects of smoking, including regulation of important biological pathways, we set out to examine this impact systematically in a sample of middle-aged African American smokers. We focused our investigation on patterns of moderation by m*MTHFR* on changes in methylation among the 909 loci previously identified by Dogan et al. (2014) as being associated with smoking status in African Americans at the genomewide level of significance.

Of particular interest was the effect of m*MTHFR* on response to smoking at G-protein coupled receptor 15 (*GPR15*), the gene containing CpG cg19859270, the probe most significantly associated with smoking identified by Beach et al. (2017). Decreased methylation of cg19859270 in smokers has been demonstrated in multiple cohorts, including subjects of European and Asian ancestry (Elliott et al., 2014). The effect appears particularly strong among smokers of African ancestry (Sun et al., 2013; Dogan et al., 2014, 2015). Work by other groups has linked smoking to increased expression of *GPR15* (Kõks et al., 2015) and shown that hypomethylation of cg19859270 in smokers directly corresponds an increased population of GPR15-expressing T cells (Bauer et al., 2015). Because *GPR15* is involved in gut homing of lymphocytes (Kim et al., 2013) and the pathophysiology of inflammatory bowel disease (Nguyen et al., 2015), it is plausible that cg19859270 hypomethylation signals an important mechanism underlying the increased risk for inflammatory disease states in smokers, particularly African Americans.

## Materials and Methods

### Informed Consent and Sample Characteristics

The consent form, procedures, and protocols pertaining to the Family and Community Health Study (FACHS) study were approved by the Institutional Review Board at the University of Iowa, the University of Georgia and Iowa State University. The individuals from FACHS who contributed their data are African-American, 62% are female (*N* = 67 male, *N* = 113 female) with an average age of 48.9 at the time of the blood draw (48.9 ± 8.6). Mean age for males (48.60 years, *SD* = 7.77) and females (48.58 years, *SD* = 9.15) did not differ.

### Collection of Blood

In each case, certified phlebotomists drew antecubital blood samples of whole blood (30 ml) from each participant and shipped it to a lab at the University of Iowa the same day for preparation. At the lab, the blood tubes were inspected to ensure anticoagulation and aliquots of blood were diluted, mononuclear cell pellets were separated from the diluted blood specimen by density-gradient centrifugation, and the mononuclear cell layer was removed from the tube using a transfer pipette, resuspended, and frozen at −80 degree C until use. Genomic DNA was prepared using a QiaAmp (Qiagen, Hilden, Germany) according to manufacturer's directions. A typical DNA yield for each mononuclear cell pellet was between 10 and 15 μg.

### Characterization of Methylation

Following the same methods described by Beach et al. (2017), methylation was assessed using the Illumina (San Diego, CA, USA) HumanMethylation450 Beadchip. Participants were randomly assigned to 12 sample “slides/chips” with groups of 8 slides being bisulfite converted in a single batch, resulting in five “batches/plates.” A replicated sample of DNA was included in each plate to aid in assessment of batch variation and to ensure correct handling of specimens. The replicated sample was examined for average correlation of beta values between plates, resulting in average correlations greater than 0.99. Prior to normalization, methylation data were filtered based on these criteria: (1) samples containing 1% of CpG sites with detection *p*-value > 0.05 were removed, (2) sites were removed if a beadcount of <3 was present in 5% of samples and (3) sites with a detection *p*-value of >0.05 in 1% of samples were removed. More than 99.76% of the 485,577 probes yielded statistically reliable data.

Prior to analyses, data were quantile normalized using the wateRmelon R package, dasen method (Pidsley et al., 2013). To create an index of methylation of MTHFR (m*MTHFR*), we utilized all loci annotated as being associated with the first exon of *MTHFR*. The resulting set of seven loci (cg02978542, cg08269394, cg12751404, cg14032528, cg23068701, cg23226134, cg23952195) were all annotated as being on the CpG island for *MTHFR* and as being promoter associated. The composite index of *MTHFR* methylation was calculated by averaging the standardized scores of the seven retained CpGs following quantile normalization. That is, m*MTHFR* = mean (zcg02978542, zcg08269394, zcg12751404, zcg14032528, zcg23068701, zcg23226134, zcg23952195). All analyses were also confirmed using a mean index of all 24 loci annotated as being associated with *MTHFR* (analyses available on request).

The other CpG probes analyzed in the current investigation were the 909 loci identified by Dogan et al. (2014) as being associated with smoking in middle age African Americans. The methylation data for the 909 loci examined in these analyses is available in Supplemental Table 4. Beta values were derived using the Illumina Genome Studio software (San Diego, CA).

### Estimation of Cell Type Proportions in Peripheral Blood

To control for the effect of varying proportions in cell types found in peripheral mononuclear cell pellets on methylation signatures (Reinius et al., 2012) we followed the procedure described by Houseman et al. (2012) through the “EstimateCellCounts” function in the minfi Bioconductor package.

### Characterization of Cigarette Smoking Status

Smoking status was measured dichotomously (1 = yes; 0 = no) based on a question at wave 5: “Did you use tobacco (cigarettes, cigars, pipe, or chewing tobacco) during the past 12 months?” In addition, at each wave of data collection, subjects were asked, “In the past month, how much did you smoke cigarettes?” Response options included 0 = none at all, 1 = less than 1 cigarette a day, 2 = 1–5 cigarettes a day, 3 = about half a pack a day, 4 = about a pack a day, 5 = about 1 and a half packs a day, and 6 = about 2 packs a day. Scores were averaged across the five waves of data collection totaling 11 years to calculate average cigarette consumption.

### Characterization of Nutritional Status

Because dietary factors including homocysteine have been shown to interact with *MTHFR* genotype in moderating the impact of environmental exposures on site-specific changes in DNA methylation (Fernandes et al., 2018; Nash et al., 2018), an index of nutritional status was constructed for each subject to be used as a covariate. Nutritional status was measured by a question: “How often do you watch what you eat (i.e., nutrition-such as eating fruits and vegetables regularly)?” Responses ranged from 1 (never) to 4 (regularly) and these values were entered into regression analyses as described below.

### Plan of Analysis

Methylation at CpGs is measured between 0 and 1 and so typically fails the assumption of having a normal distribution (Dolzhenko and Smith, 2014). Beta regressions, using the beta distribution provide a better way to handle distributions in which the dependent variable is bounded, or when it has a ceiling or floor effect, introducing a non-normal distribution for the dependent variable (Ferrari and Cribari-Neto, 2004). Accordingly, beta regression was used to examine the effect of smoking, mMTHFR and their interaction on individual CpGs. Thus, we began by running main effect beta regression models to examine main effects on methylation at each of the 909 CpGs previously identified. In a second model, an additional interaction term for smoking status^*^m*MTHFR* was included. In each model age and gender were included as controls. Results were corrected for multiple comparisons using the Bonferroni method.

Next, to examine the broader interaction effect of smoking and m*MTHFR* on indices of hyper methylated and hypo methylated loci associated with smoking, we used the OLS linear regression model available in STATA 15 was used (StataCorp, 2017). For linear regression model 1, the hyper or hypo methylation indices, respectively, were each regressed against smoking status, m*MTHFR*, gender, age, diet, and cell-type variation. For model 2, an additional interaction term for smoking status^*^m*MTHFR* was included in the regression.

## Results

The zero order correlations, means and standard deviations for all variables in the study are reported in Table 1. Approximately half (48%) of the participants reported smoking in the previous 12 months. Smoking status was not associated with the major cell types examined, nor was it associated with the m*MTHFR* index. The m*MTHFR* index was correlated with male gender *r* = 0.412 (*p* < 0.01) and with the index of hyper methylated sites *r* = −0.315 (*p* < 0.01) but not hypo methylated sites.

**Table 1:** Correlations, Means, and Standard Deviations among primary study and control variables.

**Variable**	**1**	**2**	**3**	**4**	**5**	**6**	**7**	**8**	**9**	**10**	**11**	**12**
1. Smoking status	–										
2. mMTHFR	−0.137†	–									
3. Hypo methylated index	−0.309**	−0.013	–								
4. Hyper methylated index	0.579**	−0.315**	−0.600**	–							
5. Males	0.083	0.412**	−0.112	−0.116	–						
6. Age	−0.061	−0.045	−0.109	0.005	0.065	–					
7. Diet	−0.009	0.036	−0.014	−0.081	0.144†	0.085	–				
8. CD8+T cells	0.118	−0.199**	−0.284**	0.008	−0.033	0.006	−0.038	–			
9. CD4+T cells	−0.001	−0.302**	0.138†	−0.090	−0.192**	−0.148**	0.036	0.066	–		
10. Natural killer cells	−0.064	0.029	−0.204**	0.043	0.290**	0.177**	0.038	0.126†	−0.433**	–	
11. B cells	−0.032	0.157*	−0.362**	0.118	−0.011	−0.093	0.085	−0.185*	0.045	−0.356**	–
12. Monocytes	0.068	0.164*	0.215**	−0.008	0.091	0.113	−0.038	−0.238**	−0.365**	0.162*	−0.208**	–
Mean	0.483	0.000	0.000	0.000	0.372	48.861	2.722	0.137	0.284	0.071	0.165	0.144
*SD*	0.501	0.461	0.614	0.605	0.485	8.586	1.009	0.068	0.106	0.072	0.095	0.079

*A point-biserial correlation is used to test a gender variable. Methylated indices were calculated by standardizing and averaging the beta values. This results in each index having a mean of 0*.

† p ≤ 0.10,

* *p ≤ 0.05*,

** *p ≤ 0.01 (two-tailed tests)*.

The index of the 205 loci showing enhanced hypo methylation in response to smoking among those with higher mMTHFR was correlated *r* = −0.309 (*p* = 0.000025) with smoking. The index of 66 loci showing enhanced hyper methylation in response to smoking among those with higher mMTHFR was correlated *r* = 0.579 (*p* = 1.756e-17) with smoking status. There were also significant associations between cell mixture and m*MTHFR*, suggesting the value of controlling for these as potential confounds. Nutritional status, as indicated by self-report of dietary habits, showed no correlations with m*MTHFR* or other variables.

Focusing on the 909 individual loci identified by Dogan et al. (2014) as significantly associated with smoking status at genomewide levels after Benjamini-Hochberg correction, we examined whether there were significant interactions between smoking status and m*MTHFR*, at a frequency greater than would be expected by chance. We found that *N* = 292 loci demonstrated an interaction (*p* < 0.05) between smoking status and m*MTHFR* in the prediction of methylation level, with 73 of these for loci hyper methylated in response to smoking and 219 loci hypo methylated in response to smoking. Accordingly, there was suggestive evidence for a broad impact of m*MTHFR* on the long-term methylation patterns produced by exposure to tobacco smoke.

Of additional theoretical interest was whether the patterns of amplification observed for hyper and hypo methylation would be consistent across loci, and whether they would differ for hyper and hypo methylating response to smoking. Table 2 presents results of regressing the 73 loci demonstrating hyper methylation in response to smoking on smoking, m*MTHFR*, and their interaction. As can be seen, 66 of these have a negative interaction term, indicating a consistent pattern of moderation by m*MTHFR* of the impact of smoking status. Table 3 presents the results of regressing the 219 loci with hypo methylation in response to smoking on smoking, m*MTHFR*, and their interaction. As shown in the table, 205 of these have a positive interaction term, again indicating a consistent pattern of moderation by m*MTHFR* of the impact of smoking status on hypo methylation. The strongest effect of moderation by m*MTHFR* was seen for cg19859270, with an interaction effect beta = 8.00, *p* = 9.04 × 10^−15^. The full set of moderated regression results for all 909 loci, including non-significant results, are provided in Supplemental Table 5.

**Table 2:** Beta regression of methylation levels on smoking and m*MTHFR* (Model 1), and smoking, m*MTHFR* and their interaction (Model 2), controlling for age and gender, for the 73 loci demonstrating hypermethylation associated with smoking, i.e., greater methylation among smokers than non-smokers at nominal significance levels.

	**Model 1**				**Model 2**					
	**Smo**		**mMTHFR**		**Smo**		**mMTHFR**		**Smo** **×** **mMTHFR**	
**CpG**	***b***	***p*-value**	***B***	***p*-value**	***b***	***p*-value**	***b***	***p*-value**	***b***	***p*-value**
**cg18715665**	**7.68E-02**	**4.22E-04**	**−9.24E-02**	**1.88E-04**	**7.72E-02**	**1.65E-04**	**−1.12E-02**	**7.14E-01**	**−1.97E-01**	**6.99E-06**
cg02319016	4.13E-01	1.61E-19	−1.92E-01	5.27E-04	4.14E-01	4.80E-21	−2.90E-02	6.66E-01	−3.65E-01	1.41E-04
cg09299076	5.10E-02	1.96E-02	−4.92E-03	8.47E-01	4.97E-02	1.76E-02	7.06E-02	3.14E-02	−1.68E-01	2.25E-04
cg27258878	1.55E-01	2.08E-11	−7.86E-02	5.13E-03	1.53E-01	8.51E-12	7.90E-03	8.26E-01	−1.74E-01	3.55E-04
cg08866608	1.51E-01	9.88E-05	−6.33E-02	1.99E-01	1.49E-01	8.72E-05	9.56E-02	1.29E-01	−2.91E-01	4.69E-04
cg08672695	4.52E-01	1.42E-23	−2.20E-01	7.99E-05	4.52E-01	7.17E-25	−6.17E-02	3.72E-01	−3.30E-01	5.85E-04
cg08128444	1.79E-01	3.81E-11	−6.41E-02	4.95E-02	1.78E-01	1.49E-11	2.57E-02	5.35E-01	−1.88E-01	9.52E-04
cg18040892	1.04E-01	5.72E-08	−1.38E-02	5.35E-01	1.03E-01	2.81E-08	4.09E-02	1.42E-01	−1.31E-01	1.07E-03
cg23243012	1.24E-01	1.49E-06	−7.91E-02	1.14E-02	1.22E-01	1.36E-06	−1.63E-01	2.52E-05	1.78E-01	1.08E-03
cg15281724	3.19E-01	6.01E-19	−1.51E-01	3.12E-04	3.20E-01	3.09E-20	−5.07E-02	3.23E-01	−2.44E-01	1.09E-03
cg11692477	8.39E-02	1.62E-03	−1.65E-02	6.23E-01	8.43E-02	1.27E-03	8.00E-02	6.66E-02	−1.83E-01	1.61E-03
cg01233786	9.94E-02	1.24E-03	−1.31E-01	7.59E-04	9.40E-02	1.98E-03	−2.33E-01	1.37E-06	2.04E-01	1.92E-03
cg25354716	2.60E-01	3.54E-13	−1.52E-01	4.22E-04	2.59E-01	1.08E-13	−4.45E-02	4.12E-01	−2.31E-01	2.23E-03
cg01427976	1.06E-01	1.13E-09	5.98E-03	7.77E-01	1.06E-01	6.06E-10	6.18E-02	2.24E-02	−1.12E-01	2.32E-03
cg01704976	1.41E-01	9.74E-08	3.70E-03	9.06E-01	1.40E-01	7.83E-08	8.31E-02	3.36E-02	−1.65E-01	2.33E-03
cg11253957	4.81E-02	9.94E-03	−6.14E-02	3.81E-03	4.76E-02	8.44E-03	−1.26E-02	6.37E-01	−1.16E-01	2.35E-03
cg01612443	7.03E-02	2.29E-02	3.29E-02	3.93E-01	7.17E-02	1.82E-02	1.39E-01	5.35E-03	−2.02E-01	2.36E-03
cg11553667	3.21E-01	3.16E-14	−2.32E-01	6.97E-06	3.21E-01	7.40E-15	−1.08E-01	9.27E-02	−2.68E-01	2.82E-03
cg02247175	2.84E-01	2.84E-19	4.30E-03	9.06E-01	2.83E-01	3.87E-20	8.27E-02	7.08E-02	−1.91E-01	3.81E-03
cg21078247	8.09E-02	1.10E-06	−4.73E-02	1.59E-02	8.03E-02	7.46E-07	−2.40E-03	9.24E-01	−9.80E-02	4.95E-03
cg16754643	1.86E-01	2.83E-07	−2.14E-01	4.06E-07	1.89E-01	9.75E-08	−1.27E-01	1.48E-02	−2.10E-01	5.46E-03
cg03761477	3.85E-02	3.33E-02	−1.41E-02	5.19E-01	3.82E-02	3.16E-02	3.65E-02	1.90E-01	−1.06E-01	5.58E-03
cg14681176	8.21E-02	2.24E-04	−7.18E-02	4.60E-03	8.21E-02	1.50E-04	−1.95E-02	5.41E-01	−1.26E-01	6.35E-03
cg21350392	1.06E-01	6.60E-05	−4.96E-02	1.39E-01	1.05E-01	6.92E-05	−1.28E-01	2.59E-03	1.59E-01	6.38E-03
cg15645254	8.77E-02	4.19E-09	−3.40E-02	5.26E-02	8.73E-02	2.22E-09	4.00E-03	8.58E-01	−8.54E-02	6.51E-03
cg18311516	6.58E-02	3.54E-03	−2.05E-02	4.37E-01	6.53E-02	2.95E-03	3.55E-02	2.92E-01	−1.28E-01	6.74E-03
cg12880967	7.16E-02	4.83E-03	5.07E-02	1.06E-01	7.31E-02	3.53E-03	1.28E-01	1.84E-03	−1.48E-01	6.80E-03
cg24315257	1.79E-01	1.01E-12	−1.21E-01	3.75E-05	1.79E-01	3.18E-13	−5.89E-02	1.12E-01	−1.42E-01	6.85E-03
cg18575346	1.11E-01	4.63E-07	−2.09E-02	4.12E-01	1.10E-01	2.94E-07	2.95E-02	3.55E-01	−1.22E-01	7.49E-03
cg11804350	2.92E-01	6.53E-13	−1.05E-01	2.91E-02	2.91E-01	2.29E-13	−5.72E-03	9.24E-01	−2.27E-01	7.88E-03
cg12742432	1.13E-01	5.16E-06	−6.48E-02	2.44E-02	1.13E-01	2.81E-06	−6.69E-03	8.54E-01	−1.37E-01	8.28E-03
cg24377560	1.28E-01	1.33E-07	−1.48E-01	5.54E-07	1.27E-01	1.11E-07	−8.10E-02	3.42E-02	−1.37E-01	8.38E-03
cg06431105	8.32E-02	7.02E-04	−5.23E-02	6.49E-02	8.29E-02	5.37E-04	5.99E-03	8.69E-01	−1.34E-01	9.05E-03
cg13789443	1.53E-01	3.44E-16	−1.15E-01	3.07E-07	1.53E-01	1.20E-16	−6.74E-02	1.85E-02	−1.02E-01	1.02E-02
cg25882591	2.62E-01	1.29E-26	2.42E-02	4.07E-01	2.61E-01	1.35E-27	8.37E-02	2.53E-02	−1.36E-01	1.03E-02
cg13461130	9.18E-02	2.59E-05	−7.62E-02	2.58E-03	9.23E-02	1.50E-05	−2.70E-02	3.99E-01	−1.18E-01	1.04E-02
cg16157016	5.85E-02	9.84E-03	−3.84E-02	1.39E-01	5.79E-02	8.93E-03	1.31E-02	6.93E-01	−1.20E-01	1.06E-02
cg22153062	1.22E-01	8.97E-10	−9.72E-02	4.83E-05	1.21E-01	6.58E-10	−4.55E-02	1.42E-01	−1.07E-01	1.15E-02
cg01720945	9.96E-02	3.79E-06	−5.45E-02	3.49E-02	9.92E-02	3.26E-06	−1.07E-01	9.64E-04	1.12E-01	1.30E-02
cg16830479	1.95E-01	7.41E-13	−9.57E-02	3.18E-03	1.94E-01	3.51E-13	−2.98E-02	4.71E-01	−1.43E-01	1.31E-02
cg03603505	6.56E-02	5.00E-04	−1.52E-02	4.85E-01	6.47E-02	4.53E-04	2.66E-02	3.34E-01	−9.74E-02	1.32E-02
cg15514751	3.30E-01	1.57E-29	5.41E-02	1.14E-01	3.28E-01	2.13E-30	1.21E-01	6.14E-03	−1.52E-01	1.43E-02
cg04120407	1.53E-01	2.94E-10	−9.22E-02	7.76E-04	1.54E-01	1.12E-10	−4.28E-02	2.14E-01	−1.22E-01	1.49E-02
cg07842327	1.64E-01	3.70E-05	3.28E-02	4.71E-01	1.61E-01	3.44E-05	1.15E-01	4.67E-02	−2.00E-01	1.56E-02
cg05905475	1.16E-01	1.08E-04	−6.77E-02	4.55E-02	1.15E-01	8.39E-05	−7.77E-03	8.55E-01	−1.49E-01	1.63E-02
cg01852611	1.83E-01	2.48E-11	−9.90E-02	2.42E-03	1.83E-01	1.34E-11	−3.63E-02	3.80E-01	−1.39E-01	1.64E-02
cg13566059	2.00E-01	1.73E-05	−4.00E-02	4.65E-01	1.98E-01	1.40E-05	6.58E-02	3.55E-01	−2.37E-01	1.78E-02
cg16280946	8.78E-02	5.69E-06	−9.85E-02	1.69E-05	8.75E-02	4.28E-06	−5.55E-02	5.74E-02	−9.53E-02	1.98E-02
cg24865495	1.42E-01	2.98E-10	−4.51E-02	8.75E-02	1.42E-01	1.30E-10	2.04E-03	9.52E-01	−1.10E-01	2.14E-02
cg14195606	9.42E-02	1.17E-06	−1.19E-01	2.02E-07	9.44E-02	7.43E-07	−7.75E-02	7.51E-03	−9.43E-02	2.16E-02
cg24162465	8.98E-02	1.23E-04	−6.17E-02	2.45E-02	9.01E-02	8.66E-05	−1.14E-02	7.48E-01	−1.12E-01	2.37E-02
cg11168432	9.05E-02	1.47E-03	−7.11E-03	8.22E-01	8.86E-02	1.50E-03	4.43E-02	2.61E-01	−1.31E-01	2.41E-02
cg19165344	8.39E-02	2.83E-05	−7.58E-02	1.38E-03	8.36E-02	2.27E-05	−3.28E-02	2.80E-01	−9.50E-02	2.50E-02
cg02569236	8.59E-02	4.64E-04	6.36E-02	3.72E-02	8.69E-02	3.55E-04	1.25E-01	1.88E-03	−1.19E-01	2.62E-02
cg15369419	1.88E-01	1.30E-08	−9.63E-02	1.10E-02	1.88E-01	6.33E-09	−3.52E-02	4.53E-01	−1.52E-01	2.63E-02
cg00068377	9.34E-02	4.63E-04	−2.61E-02	4.25E-01	9.38E-02	3.97E-04	−8.25E-02	4.01E-02	1.27E-01	2.63E-02
cg16385237	6.49E-02	1.25E-02	2.06E-03	9.48E-01	6.47E-02	1.20E-02	6.42E-02	1.19E-01	−1.23E-01	2.69E-02
cg13762887	1.05E-01	7.84E-09	−9.32E-03	6.62E-01	1.04E-01	4.97E-09	2.73E-02	3.16E-01	−8.47E-02	2.80E-02
cg20891060	1.42E-01	3.21E-08	9.14E-02	2.82E-03	1.41E-01	2.86E-08	1.47E-01	2.12E-04	−1.21E-01	2.82E-02
cg06056514	5.22E-02	5.99E-03	−3.17E-02	1.50E-01	5.19E-02	5.44E-03	5.84E-03	8.36E-01	−8.66E-02	2.99E-02
cg01341751	1.01E-01	1.04E-04	−1.29E-01	3.40E-05	9.89E-02	1.30E-04	−1.82E-01	2.41E-06	1.18E-01	3.09E-02
cg01337429	8.94E-02	9.73E-06	−2.11E-03	9.28E-01	8.86E-02	8.32E-06	3.72E-02	2.12E-01	−9.06E-02	3.14E-02
cg09917026	1.75E-01	1.06E-13	−2.10E-02	4.39E-01	1.75E-01	4.23E-14	2.18E-02	5.23E-01	−1.06E-01	3.29E-02
cg06557376	7.51E-02	4.40E-03	−2.10E-02	4.89E-01	7.43E-02	4.17E-03	2.99E-02	4.44E-01	−1.17E-01	3.44E-02
cg10553894	7.46E-02	1.19E-04	−3.65E-02	1.04E-01	7.42E-02	1.03E-04	−8.07E-04	9.77E-01	−8.42E-02	3.84E-02
cg04792227	1.02E-01	2.77E-02	−5.08E-02	3.80E-01	1.00E-01	2.97E-02	5.67E-02	4.54E-01	−2.09E-01	4.01E-02
cg24807761	8.31E-02	7.31E-04	−1.08E-01	3.56E-04	8.18E-02	8.26E-04	−1.57E-01	3.20E-05	1.09E-01	4.20E-02
cg25371449	6.48E-02	2.23E-03	−6.06E-02	1.23E-02	6.48E-02	1.93E-03	−2.30E-02	4.53E-01	−8.92E-02	4.27E-02
cg25987208	1.56E-01	2.57E-06	4.11E-02	3.00E-01	1.56E-01	2.17E-06	1.08E-01	3.42E-02	−1.41E-01	4.43E-02
cg14941559	1.09E-01	2.07E-09	−1.78E-02	4.04E-01	1.08E-01	1.56E-09	1.65E-02	5.46E-01	−7.69E-02	4.57E-02
cg26607002	1.04E-01	8.69E-08	−4.34E-02	5.87E-02	1.04E-01	7.37E-08	−6.02E-03	8.39E-01	−8.22E-02	4.65E-02
cg17267720	1.32E-01	4.63E-06	−7.22E-02	3.57E-02	1.31E-01	4.11E-06	−1.63E-02	7.13E-01	−1.21E-01	4.89E-02
cg02933375	1.19E-01	3.60E-07	−6.32E-02	1.80E-02	1.19E-01	2.47E-07	−2.46E-02	4.63E-01	−9.52E-02	4.98E-02

*Values bolded indicate loci significantly predicted by the interaction of smoking and mMTHFR after Bonferroni correction for 909 tests (p < 5.500-05)*.

**Table 3:** Beta regression on smoking and mMTHFR (Model 1) controlling for age and gender, and smoking, mMTHFR and their interaction (Model 2) controlling for age and gender, for the 219 loci demonstrating hypomethylation associated with smoking, i.e., significantly lower methylation among smokers than non-smokers at nominal significance levels.

	**Model 1**				**Model 2**					
	**Smo**		**mMTHFR**		**Smo**		**mMTHFR**		**Smo** **×** **mMTHFR**	
**CpG**	***b***	***p*-value**	***B***	***p*-value**	***b***	***p*-value**	***b***	***p*-value**	***b***	***p*-value**
**cg19859270**	**−4.46E-01**	**9.35E-16**	**−2.56E-01**	**6.16E-04**	**−4.99E-01**	**8.35E-23**	**−7.20E-01**	**1.49E-22**	**8.00E-01**	**9.04E-16**
**cg19614811**	**−2.07E-01**	**1.04E-04**	**−1.67E-01**	**1.88E-02**	**−2.30E-01**	**5.35E-06**	**−5.15E-01**	**2.38E-11**	**6.36E-01**	**1.77E-09**
**cg23313266**	**−5.74E-02**	**4.63E-02**	**−1.30E-01**	**2.22E-04**	**−6.14E-02**	**2.11E-02**	**−2.94E-01**	**6.97E-13**	**3.38E-01**	**2.30E-09**
**cg03038850**	**−5.90E-02**	**1.03E-02**	**−5.23E-02**	**6.22E-02**	**−6.00E-02**	**4.80E-03**	**−1.84E-01**	**1.97E-08**	**2.70E-01**	**2.55E-09**
**cg02706575**	**−5.91E-02**	**6.05E-02**	**−5.16E-02**	**1.99E-01**	**−6.39E-02**	**2.98E-02**	**−2.45E-01**	**1.94E-07**	**3.70E-01**	**6.12E-09**
**cg05633605**	**−8.30E-02**	**9.08E-02**	**−1.37E-01**	**2.74E-02**	**−9.47E-02**	**4.02E-02**	**−4.14E-01**	**1.78E-09**	**5.55E-01**	**7.63E-09**
**cg19655032**	**−3.64E-02**	**7.07E-02**	**−3.84E-02**	**1.22E-01**	**−3.72E-02**	**4.72E-02**	**−1.51E-01**	**3.73E-07**	**2.27E-01**	**2.15E-08**
**cg04880611**	**−3.64E-02**	**1.88E-01**	**−3.28E-02**	**3.50E-01**	**−3.92E-02**	**1.31E-01**	**−1.95E-01**	**3.04E-06**	**3.13E-01**	**2.68E-08**
**cg09043104**	**−9.14E-02**	**2.03E-03**	**−1.15E-01**	**1.72E-03**	**−9.62E-02**	**5.32E-04**	**−2.77E-01**	**1.57E-10**	**3.26E-01**	**3.98E-08**
**cg16149164**	**−8.53E-02**	**1.31E-02**	**−4.66E-02**	**2.60E-01**	**−8.44E-02**	**8.41E-03**	**−2.20E-01**	**7.24E-06**	**3.70E-01**	**5.69E-08**
**cg16922869**	**−5.53E-02**	**9.59E-02**	**−2.85E-02**	**4.98E-01**	**−5.80E-02**	**6.39E-02**	**−2.18E-01**	**1.28E-05**	**3.65E-01**	**6.40E-08**
**cg21646084**	**−4.40E-02**	**5.28E-02**	**−8.65E-02**	**2.10E-03**	**−4.58E-02**	**3.17E-02**	**−2.09E-01**	**5.00E-10**	**2.48E-01**	**7.19E-08**
**cg19069039**	**−3.02E-02**	**1.83E-01**	**−7.31E-04**	**9.79E-01**	**−3.05E-02**	**1.51E-01**	**−1.26E-01**	**2.19E-04**	**2.48E-01**	**8.37E-08**
**cg00959749**	**−1.20E-01**	**4.01E-03**	**−1.84E-01**	**2.84E-04**	**−1.26E-01**	**1.34E-03**	**−3.95E-01**	**3.06E-11**	**4.40E-01**	**8.83E-08**
**cg14191885**	**−6.79E-02**	**4.31E-02**	**−1.19E-02**	**7.76E-01**	**−6.90E-02**	**2.92E-02**	**−1.94E-01**	**1.11E-04**	**3.58E-01**	**1.45E-07**
**cg06648782**	**−7.47E-02**	**2.15E-03**	**−2.65E-02**	**3.77E-01**	**−7.60E-02**	**9.29E-04**	**−1.60E-01**	**1.31E-05**	**2.56E-01**	**1.94E-07**
**cg00831247**	**−5.41E-02**	**2.64E-02**	**−1.65E-01**	**3.88E-08**	**−5.80E-02**	**1.15E-02**	**−2.92E-01**	**5.89E-16**	**2.55E-01**	**2.12E-07**
**cg05079547**	**−9.54E-02**	**2.67E-02**	**−4.38E-02**	**4.10E-01**	**−9.64E-02**	**1.76E-02**	**−2.61E-01**	**2.79E-05**	**4.45E-01**	**2.42E-07**
**cg15267250**	**−2.88E-02**	**2.14E-01**	**−6.78E-02**	**1.82E-02**	**−3.04E-02**	**1.65E-01**	**−1.88E-01**	**6.26E-08**	**2.42E-01**	**3.38E-07**
**cg27063327**	**−3.25E-02**	**2.04E-01**	**−7.49E-02**	**1.98E-02**	**−3.50E-02**	**1.50E-01**	**−2.10E-01**	**5.21E-08**	**2.67E-01**	**3.62E-07**
**cg14303616**	**−1.40E-02**	**4.07E-01**	**3.58E-02**	**8.39E-02**	**−1.29E-02**	**4.18E-01**	**−4.94E-02**	**5.00E-02**	**1.75E-01**	**3.90E-07**
**cg00642607**	**−6.13E-02**	**1.46E-02**	**−3.26E-02**	**2.86E-01**	**−6.13E-02**	**9.95E-03**	**−1.54E-01**	**2.30E-05**	**2.52E-01**	**5.95E-07**
**cg10216717**	**−8.70E-02**	**9.15E-04**	**−8.26E-02**	**9.69E-03**	**−8.82E-02**	**3.72E-04**	**−2.11E-01**	**4.89E-08**	**2.64E-01**	**6.48E-07**
**cg24263998**	**−5.33E-02**	**3.76E-02**	**−6.91E-02**	**3.39E-02**	**−5.75E-02**	**1.85E-02**	**−2.10E-01**	**1.63E-07**	**2.64E-01**	**7.48E-07**
**cg24876404**	**−6.86E-02**	**1.11E-01**	**−2.98E-02**	**5.71E-01**	**−6.83E-02**	**9.41E-02**	**−2.30E-01**	**1.99E-04**	**4.22E-01**	**1.05E-06**
**cg04294388**	**−3.20E-02**	**2.58E-01**	**5.33E-03**	**8.81E-01**	**−3.23E-02**	**2.30E-01**	**−1.39E-01**	**1.27E-03**	**2.85E-01**	**1.09E-06**
**cg27356115**	**−1.71E-01**	**6.93E-05**	**−9.25E-02**	**6.71E-02**	**−1.68E-01**	**3.20E-05**	**−2.80E-01**	**4.67E-06**	**4.19E-01**	**1.10E-06**
**cg23473088**	**−4.74E-02**	**4.28E-02**	**−1.25E-02**	**6.65E-01**	**−4.71E-02**	**3.38E-02**	**−1.26E-01**	**2.84E-04**	**2.32E-01**	**1.12E-06**
**cg23674788**	**−3.81E-02**	**5.91E-02**	**−8.90E-04**	**9.72E-01**	**−3.78E-02**	**4.79E-02**	**−1.02E-01**	**8.93E-04**	**2.02E-01**	**1.15E-06**
**cg15110219**	**−9.15E-03**	**7.41E-01**	**6.99E-02**	**4.54E-02**	**−7.12E-03**	**7.87E-01**	**−7.35E-02**	**8.48E-02**	**2.80E-01**	**1.18E-06**
**cg11779204**	**−4.36E-02**	**6.77E-02**	**3.70E-02**	**2.13E-01**	**−4.25E-02**	**6.07E-02**	**−8.19E-02**	**2.34E-02**	**2.39E-01**	**1.19E-06**
**cg19834585**	**−3.54E-02**	**1.57E-01**	**−1.70E-03**	**9.56E-01**	**−3.50E-02**	**1.39E-01**	**−1.24E-01**	**1.02E-03**	**2.47E-01**	**1.52E-06**
**cg18731202**	**−1.42E-01**	**1.15E-04**	**7.37E-02**	**1.14E-01**	**−1.41E-01**	**6.44E-05**	**−1.27E-01**	**2.81E-02**	**3.66E-01**	**1.60E-06**
**cg18985133**	**−2.07E-02**	**3.15E-01**	**−3.39E-02**	**1.85E-01**	**−2.18E-02**	**2.66E-01**	**−1.37E-01**	**1.34E-05**	**2.03E-01**	**1.76E-06**
**cg03168749**	**−5.39E-02**	**2.48E-02**	**2.90E-02**	**3.30E-01**	**−5.33E-02**	**1.98E-02**	**−8.80E-02**	**1.58E-02**	**2.34E-01**	**2.38E-06**
**cg14905634**	**−1.03E-01**	**1.94E-02**	**−2.95E-02**	**5.78E-01**	**−1.02E-01**	**1.46E-02**	**−2.25E-01**	**3.94E-04**	**4.16E-01**	**2.62E-06**
**cg03662571**	**−5.15E-02**	**1.61E-02**	**9.25E-03**	**7.27E-01**	**−5.10E-02**	**1.23E-02**	**−9.58E-02**	**3.39E-03**	**2.07E-01**	**2.80E-06**
**cg11649016**	**−5.71E-02**	**2.29E-03**	**−1.43E-02**	**5.35E-01**	**−5.70E-02**	**1.39E-03**	**−1.05E-01**	**2.36E-04**	**1.80E-01**	**3.05E-06**
**cg03651715**	**−6.48E-02**	**4.24E-02**	**−6.29E-02**	**9.68E-02**	**−6.33E-02**	**3.65E-02**	**−1.98E-01**	**1.50E-05**	**2.96E-01**	**3.89E-06**
**cg20752695**	**−8.07E-02**	**7.00E-03**	**−4.78E-02**	**1.93E-01**	**−8.16E-02**	**4.23E-03**	**−1.87E-01**	**3.03E-05**	**2.81E-01**	**4.39E-06**
**cg03930929**	**−1.16E-01**	**4.46E-02**	**2.52E-03**	**9.73E-01**	**−1.19E-01**	**3.16E-02**	**−2.76E-01**	**1.27E-03**	**5.42E-01**	**4.73E-06**
**cg26270695**	**−1.12E-01**	**1.63E-03**	**−6.05E-02**	**1.76E-01**	**−1.16E-01**	**7.23E-04**	**−2.31E-01**	**1.95E-05**	**3.35E-01**	**4.99E-06**
**cg14430629**	**−2.21E-02**	**1.85E-01**	**2.82E-03**	**8.90E-01**	**−2.17E-02**	**1.71E-01**	**−7.48E-02**	**3.16E-03**	**1.56E-01**	**5.44E-06**
**cg19889666**	**−2.76E-02**	**1.32E-01**	**2.24E-02**	**3.18E-01**	**−2.66E-02**	**1.28E-01**	**−6.29E-02**	**2.41E-02**	**1.72E-01**	**5.74E-06**
**cg16314263**	**−6.45E-02**	**7.37E-03**	**−3.74E-02**	**2.02E-01**	**−6.47E-02**	**4.96E-03**	**−1.44E-01**	**5.89E-05**	**2.20E-01**	**7.73E-06**
**cg16062877**	**−8.83E-02**	**6.26E-03**	**−5.18E-02**	**1.87E-01**	**−8.88E-02**	**3.97E-03**	**−1.95E-01**	**4.82E-05**	**2.95E-01**	**7.79E-06**
**cg18396789**	**−5.38E-02**	**1.83E-01**	**1.35E-02**	**7.94E-01**	**−5.54E-02**	**1.55E-01**	**−1.84E-01**	**3.33E-03**	**3.79E-01**	**7.82E-06**
**cg00608779**	**−6.41E-02**	**3.85E-02**	**2.60E-02**	**5.03E-01**	**−6.41E-02**	**3.09E-02**	**−1.18E-01**	**1.34E-02**	**2.84E-01**	**1.12E-05**
**cg14254999**	**−1.70E-01**	**1.31E-05**	**−4.02E-03**	**9.35E-01**	**−1.72E-01**	**4.60E-06**	**−1.96E-01**	**1.47E-03**	**3.54E-01**	**1.46E-05**
**cg03333116**	**−4.39E-02**	**7.44E-03**	**2.20E-02**	**2.72E-01**	**−4.29E-02**	**6.40E-03**	**−4.95E-02**	**4.66E-02**	**1.46E-01**	**1.71E-05**
**cg09934692**	**−5.31E-02**	**3.43E-02**	**−8.56E-02**	**6.74E-03**	**−5.66E-02**	**1.93E-02**	**−2.03E-01**	**2.91E-07**	**2.24E-01**	**2.31E-05**
**cg02423618**	**−4.56E-02**	**1.31E-01**	**−4.98E-02**	**1.70E-01**	**−4.52E-02**	**1.18E-01**	**−1.72E-01**	**1.05E-04**	**2.60E-01**	**2.42E-05**
**cg18115235**	**−8.21E-02**	**2.88E-02**	**1.22E-02**	**7.91E-01**	**−8.16E-02**	**2.38E-02**	**−1.47E-01**	**9.22E-03**	**3.24E-01**	**2.91E-05**
**cg17632028**	**−6.50E-02**	**1.27E-03**	**−2.21E-02**	**3.71E-01**	**−6.51E-02**	**7.90E-04**	**−1.08E-01**	**4.26E-04**	**1.75E-01**	**3.04E-05**
**cg19418648**	**−5.57E-02**	**1.80E-02**	**1.10E-02**	**7.02E-01**	**−5.49E-02**	**1.50E-02**	**−8.76E-02**	**1.41E-02**	**2.03E-01**	**3.38E-05**
**cg25538883**	**−5.46E-02**	**1.87E-02**	**3.49E-02**	**2.24E-01**	**−5.37E-02**	**1.62E-02**	**−6.50E-02**	**7.06E-02**	**2.00E-01**	**4.09E-05**
**cg18016288**	**−1.11E-01**	**2.20E-03**	**3.91E-02**	**3.65E-01**	**−1.09E-01**	**1.67E-03**	**−1.01E-01**	**5.70E-02**	**3.03E-01**	**4.15E-05**
**cg26061593**	**−6.49E-02**	**1.34E-02**	**−6.97E-04**	**9.83E-01**	**−6.40E-02**	**1.13E-02**	**−1.08E-01**	**6.17E-03**	**2.21E-01**	**4.36E-05**
**cg07500957**	**−6.39E-02**	**5.57E-03**	**1.63E-02**	**5.65E-01**	**−6.35E-02**	**4.27E-03**	**−8.16E-02**	**2.15E-02**	**1.95E-01**	**4.86E-05**
**cg18450625**	**−1.53E-01**	**3.13E-04**	**6.37E-02**	**2.26E-01**	**−1.51E-01**	**2.36E-04**	**−1.16E-01**	**7.39E-02**	**3.55E-01**	**5.40E-05**
cg10888878	−1.58E-02	4.09E-01	−3.26E-02	1.58E-01	−1.59E-02	3.87E-01	−1.09E-01	1.44E-04	1.58E-01	5.96E-05
cg25147684	−6.01E-02	3.42E-03	−4.43E-02	7.72E-02	−6.05E-02	2.22E-03	−1.30E-01	3.88E-05	1.71E-01	6.09E-05
cg19153095	−7.29E-02	1.01E-01	4.60E-02	4.22E-01	−7.34E-02	8.91E-02	−1.54E-01	2.82E-02	3.79E-01	6.26E-05
cg18748085	−6.13E-02	3.19E-02	6.00E-03	8.67E-01	−6.22E-02	2.45E-02	−1.20E-01	8.12E-03	2.40E-01	6.66E-05
cg14878128	−1.29E-01	1.25E-02	−9.51E-03	8.83E-01	−1.31E-01	9.15E-03	−2.27E-01	3.95E-03	4.27E-01	6.80E-05
cg20668718	−1.17E-01	1.07E-02	−5.80E-02	3.20E-01	−1.21E-01	6.57E-03	−2.61E-01	2.69E-04	3.82E-01	7.18E-05
cg15386853	−4.27E-02	3.07E-02	−4.06E-02	9.35E-02	−4.31E-02	2.40E-02	−1.22E-01	6.25E-05	1.64E-01	7.37E-05
cg27130993	−8.39E-02	4.67E-02	−2.08E-02	6.83E-01	−8.27E-02	4.21E-02	−1.81E-01	3.61E-03	3.43E-01	7.65E-05
cg22851561	−1.77E-01	1.55E-06	−6.65E-02	1.16E-01	−1.73E-01	9.08E-07	−1.89E-01	2.18E-04	2.91E-01	8.01E-05
cg01769354	−4.41E-02	6.18E-03	−2.96E-02	1.24E-01	−4.33E-02	5.17E-03	−8.98E-02	2.04E-04	1.28E-01	1.29E-04
cg11648740	−4.26E-02	1.02E-01	2.24E-02	4.91E-01	−4.27E-02	9.11E-02	−8.48E-02	3.86E-02	2.11E-01	1.37E-04
cg05740739	−1.29E-01	4.17E-05	−2.26E-02	5.51E-01	−1.29E-01	2.29E-05	−1.42E-01	2.47E-03	2.46E-01	1.37E-04
cg11870561	−6.57E-02	1.86E-02	−3.53E-02	3.13E-01	−6.69E-02	1.37E-02	−1.52E-01	6.13E-04	2.24E-01	1.59E-04
cg21734175	−9.41E-02	6.85E-04	−3.59E-02	2.78E-01	−9.32E-02	4.97E-04	−1.36E-01	9.94E-04	2.14E-01	1.80E-04
cg24340655	−5.14E-02	7.04E-02	8.99E-02	1.12E-02	−4.97E-02	7.20E-02	−2.56E-02	5.69E-01	2.24E-01	2.03E-04
cg17731547	−5.57E-02	5.65E-02	4.72E-02	1.89E-01	−5.46E-02	5.37E-02	−6.43E-02	1.53E-01	2.26E-01	2.23E-04
cg25468907	−1.38E-02	5.14E-01	−1.63E-02	5.35E-01	−1.39E-02	5.00E-01	−9.88E-02	2.96E-03	1.65E-01	2.56E-04
cg11580351	−1.41E-01	6.27E-04	4.29E-02	3.98E-01	−1.40E-01	4.64E-04	−1.14E-01	7.32E-02	3.11E-01	3.06E-04
cg07065737	−2.38E-02	2.14E-01	−6.63E-03	7.77E-01	−2.36E-02	2.05E-01	−7.82E-02	8.19E-03	1.46E-01	3.06E-04
cg01209150	−8.50E-02	3.25E-02	−5.17E-03	9.19E-01	−8.82E-02	2.33E-02	−1.72E-01	7.47E-03	3.07E-01	3.13E-04
cg14412794	−1.64E-01	2.55E-07	−2.61E-02	5.04E-01	−1.65E-01	1.21E-07	−1.49E-01	2.51E-03	2.37E-01	3.27E-04
cg18007641	−1.66E-01	2.17E-04	−2.51E-02	6.47E-01	−1.67E-01	1.43E-04	−1.91E-01	5.35E-03	3.35E-01	3.38E-04
cg27345534	−6.50E-02	5.14E-02	4.84E-02	2.44E-01	−6.44E-02	4.78E-02	−8.23E-02	1.18E-01	2.53E-01	3.41E-04
cg20330023	−1.18E-02	4.33E-01	2.98E-02	1.04E-01	−1.10E-02	4.54E-01	−2.48E-02	2.83E-01	1.13E-01	3.45E-04
cg14414124	−8.56E-02	3.18E-04	1.08E-01	1.43E-04	−8.63E-02	1.80E-04	1.89E-01	9.97E-08	−1.77E-01	3.47E-04
cg15034413	−6.71E-02	9.82E-04	4.43E-02	8.05E-02	−6.56E-02	9.42E-04	−3.41E-02	2.91E-01	1.55E-01	3.65E-04
cg22092397	−5.34E-02	4.66E-02	3.15E-02	3.33E-01	−5.26E-02	4.33E-02	−6.45E-02	1.15E-01	1.99E-01	4.04E-04
cg18327772	−4.35E-02	8.75E-02	3.27E-02	2.93E-01	−4.25E-02	8.64E-02	−6.14E-02	1.19E-01	1.88E-01	4.15E-04
cg12720459	−7.31E-02	3.10E-05	2.00E-01	5.41E-22	−7.05E-02	3.63E-05	2.55E-01	3.67E-24	−1.27E-01	4.24E-04
cg13502545	−5.95E-02	4.60E-03	−4.23E-02	1.03E-01	−5.95E-02	3.58E-03	−1.21E-01	2.37E-04	1.56E-01	4.39E-04
cg21223803	−1.73E-02	3.65E-01	2.31E-02	3.24E-01	−1.65E-02	3.75E-01	−4.69E-02	1.16E-01	1.42E-01	4.82E-04
cg21696055	−2.02E-02	2.34E-01	1.96E-02	3.48E-01	−1.96E-02	2.37E-01	−4.26E-02	1.09E-01	1.26E-01	5.01E-04
cg07972458	−8.09E-02	7.25E-03	−7.18E-02	4.18E-02	−7.89E-02	6.81E-03	−1.67E-01	1.40E-04	2.14E-01	5.50E-04
cg08914271	−5.52E-02	4.23E-04	1.10E-04	9.95E-01	−5.44E-02	3.44E-04	−5.27E-02	2.54E-02	1.12E-01	5.61E-04
cg20536794	−4.58E-02	7.93E-02	7.13E-02	3.04E-02	−4.51E-02	7.67E-02	−3.10E-02	4.68E-01	1.93E-01	6.39E-04
cg19023320	−2.24E-02	1.81E-01	1.61E-02	4.25E-01	−2.16E-02	1.84E-01	−4.05E-02	1.09E-01	1.19E-01	6.41E-04
cg11543196	−7.36E-02	3.70E-03	−7.63E-02	1.48E-02	−7.57E-02	2.29E-03	−1.72E-01	1.86E-05	1.83E-01	6.59E-04
cg07468782	−8.57E-02	4.92E-05	−5.03E-02	4.68E-02	−8.55E-02	3.10E-05	−1.22E-01	1.39E-04	1.50E-01	6.66E-04
cg01938570	−6.90E-02	1.71E-03	5.66E-02	3.66E-02	−6.75E-02	1.70E-03	−2.26E-02	5.11E-01	1.58E-01	6.76E-04
cg26698819	−5.91E-02	2.37E-02	3.74E-02	2.45E-01	−5.85E-02	2.18E-02	−5.68E-02	1.66E-01	1.87E-01	7.21E-04
cg05066621	−4.35E-02	7.11E-02	5.69E-02	5.60E-02	−4.23E-02	7.21E-02	−2.96E-02	4.35E-01	1.73E-01	7.35E-04
cg16361253	−5.47E-02	4.52E-03	−2.17E-02	3.49E-01	−5.45E-02	3.66E-03	−8.77E-02	2.94E-03	1.36E-01	7.42E-04
cg22852353	−9.13E-02	4.57E-03	−2.62E-02	5.18E-01	−9.22E-02	3.42E-03	−1.45E-01	4.73E-03	2.32E-01	7.71E-04
cg08532673	−6.29E-02	1.86E-03	6.25E-03	8.02E-01	−6.24E-02	1.55E-03	−6.49E-02	4.01E-02	1.44E-01	7.88E-04
cg09333325	−4.45E-02	4.41E-02	−4.38E-02	1.15E-01	−4.54E-02	3.54E-02	−1.27E-01	3.92E-04	1.60E-01	8.11E-04
cg24311704	−4.62E-02	3.07E-02	3.96E-02	1.31E-01	−4.55E-02	2.90E-02	−3.52E-02	2.92E-01	1.51E-01	8.82E-04
cg01785490	−5.62E-02	1.10E-01	1.61E-02	7.17E-01	−5.64E-02	1.02E-01	−1.15E-01	4.16E-02	2.51E-01	8.99E-04
cg24804436	−3.59E-02	1.40E-01	9.55E-03	7.52E-01	−3.59E-02	1.31E-01	−7.74E-02	4.43E-02	1.72E-01	9.08E-04
cg02995567	−9.92E-02	8.76E-07	4.34E-02	6.19E-02	−9.96E-02	3.13E-07	−1.42E-02	6.25E-01	1.38E-01	9.34E-04
cg04885881	−2.05E-01	6.04E-07	−1.33E-01	5.01E-03	−2.00E-01	4.81E-07	−2.51E-01	2.04E-05	2.78E-01	9.61E-04
cg16851858	−4.48E-02	2.98E-03	2.16E-02	2.33E-01	−4.41E-02	2.72E-03	−2.88E-02	2.12E-01	1.04E-01	9.82E-04
cg26057840	−1.13E-01	1.75E-04	5.84E-02	1.10E-01	−1.11E-01	1.67E-04	−4.36E-02	3.47E-01	2.06E-01	1.07E-03
cg26456259	−6.24E-02	1.04E-02	8.15E-02	5.87E-03	−6.07E-02	1.07E-02	−8.56E-04	9.82E-01	1.67E-01	1.09E-03
cg00911962	−1.11E-01	3.13E-02	−6.73E-02	2.93E-01	−1.17E-01	2.11E-02	−2.44E-01	2.09E-03	3.51E-01	1.11E-03
cg12752325	−7.13E-02	1.71E-02	9.67E-03	7.90E-01	−7.07E-02	1.53E-02	−8.92E-02	5.24E-02	2.03E-01	1.24E-03
cg00833661	−5.07E-02	4.06E-02	6.23E-02	4.15E-02	−4.92E-02	4.27E-02	−2.42E-02	5.36E-01	1.69E-01	1.27E-03
cg03621406	−1.05E-01	5.68E-05	2.37E-01	2.10E-14	−9.95E-02	9.75E-05	3.14E-01	9.16E-17	−1.73E-01	1.35E-03
cg26440142	−4.13E-02	4.60E-02	3.81E-02	1.34E-01	−4.06E-02	4.50E-02	−3.15E-02	3.29E-01	1.41E-01	1.35E-03
cg03502236	−7.92E-02	3.39E-04	−4.90E-02	6.56E-02	−7.93E-02	2.39E-04	−1.21E-01	3.62E-04	1.48E-01	1.38E-03
cg08099570	−3.52E-02	3.63E-02	−1.54E-02	4.44E-01	−3.47E-02	3.42E-02	−6.87E-02	7.33E-03	1.12E-01	1.44E-03
cg18910630	−6.65E-02	1.27E-03	8.14E-02	1.29E-03	−6.51E-02	1.25E-03	1.24E-02	7.02E-01	1.40E-01	1.44E-03
cg14651082	−6.11E-02	5.43E-04	9.13E-03	6.70E-01	−6.05E-02	4.46E-04	−4.87E-02	7.51E-02	1.19E-01	1.45E-03
cg23817637	−2.25E-01	6.34E-05	−1.70E-01	1.10E-02	−2.25E-01	4.18E-05	−3.45E-01	4.80E-05	3.72E-01	1.48E-03
cg23170535	−4.20E-02	5.76E-02	2.79E-02	3.12E-01	−4.19E-02	5.34E-02	−5.02E-02	1.60E-01	1.51E-01	1.53E-03
cg25840536	−4.90E-02	1.08E-03	−2.41E-02	1.81E-01	−4.87E-02	8.79E-04	−7.18E-02	1.75E-03	9.93E-02	1.59E-03
cg12661610	−4.64E-02	2.36E-03	−3.29E-02	7.33E-02	−4.63E-02	1.88E-03	−8.16E-02	5.02E-04	1.01E-01	1.64E-03
cg00421612	−6.81E-02	3.82E-02	2.62E-02	5.17E-01	−6.66E-02	3.84E-02	−8.06E-02	1.14E-01	2.19E-01	1.69E-03
cg07346171	−1.42E-01	3.71E-05	6.85E-03	8.70E-01	−1.40E-01	3.22E-05	−1.07E-01	4.39E-02	2.24E-01	1.80E-03
cg14778576	−4.23E-02	2.45E-02	−7.52E-02	8.84E-04	−4.31E-02	1.95E-02	−1.35E-01	2.77E-06	1.22E-01	1.99E-03
cg15756928	−8.29E-02	1.77E-04	−3.52E-02	1.88E-01	−8.24E-02	1.41E-04	−1.05E-01	1.98E-03	1.43E-01	2.03E-03
cg00486022	−3.58E-02	1.00E-01	9.79E-02	2.87E-04	−3.41E-02	1.10E-01	2.47E-02	4.80E-01	1.44E-01	2.11E-03
cg12584590	−4.71E-02	1.52E-02	4.82E-02	4.00E-02	−4.59E-02	1.58E-02	−1.25E-02	6.75E-01	1.25E-01	2.12E-03
cg08240592	−2.51E-02	8.32E-02	−4.43E-03	8.00E-01	−2.46E-02	8.21E-02	−4.87E-02	2.80E-02	9.31E-02	2.26E-03
cg14429979	−7.36E-02	2.08E-02	−1.18E-01	2.89E-03	−7.76E-02	1.33E-02	−2.25E-01	9.00E-06	2.07E-01	2.37E-03
cg17413252	−6.85E-02	7.32E-03	−2.51E-02	4.22E-01	−6.85E-02	6.22E-03	−1.06E-01	7.63E-03	1.64E-01	2.43E-03
cg21860285	−5.57E-02	5.96E-04	−7.90E-02	4.88E-05	−5.59E-02	4.28E-04	−1.29E-01	2.24E-07	1.03E-01	2.47E-03
cg10542127	−3.91E-02	3.95E-02	5.41E-02	1.65E-02	−3.84E-02	3.82E-02	−1.58E-03	9.56E-01	1.20E-01	2.56E-03
cg14487577	−9.78E-02	1.45E-02	6.79E-02	1.85E-01	−9.85E-02	1.26E-02	−7.57E-02	2.56E-01	2.63E-01	2.62E-03
cg07589519	−4.26E-02	2.97E-03	6.21E-02	2.91E-04	−4.13E-02	3.30E-03	1.94E-02	3.73E-01	9.00E-02	2.70E-03
cg09580249	−4.18E-02	1.33E-02	4.82E-02	1.94E-02	−4.08E-02	1.39E-02	−5.03E-03	8.49E-01	1.08E-01	2.70E-03
cg12617684	−6.72E-02	2.28E-02	3.52E-02	3.32E-01	−6.64E-02	2.18E-02	−5.77E-02	2.12E-01	1.88E-01	2.75E-03
cg19623519	−2.52E-02	5.93E-02	1.33E-02	4.10E-01	−2.46E-02	6.06E-02	−2.70E-02	1.89E-01	8.44E-02	2.87E-03
cg16608731	−6.60E-02	9.40E-03	6.72E-02	3.03E-02	−6.42E-02	1.01E-02	−9.76E-03	8.04E-01	1.59E-01	3.10E-03
cg03793872	−6.18E-02	2.06E-02	5.29E-02	1.10E-01	−6.08E-02	2.03E-02	−3.21E-02	4.49E-01	1.69E-01	3.14E-03
cg25490241	−7.10E-02	1.99E-02	1.97E-02	5.95E-01	−6.98E-02	2.03E-02	−7.47E-02	1.15E-01	1.87E-01	3.40E-03
cg00690392	−4.75E-02	2.65E-03	1.98E-02	3.00E-01	−4.69E-02	2.51E-03	−2.69E-02	2.67E-01	9.73E-02	3.47E-03
cg18998938	−1.43E-01	2.24E-05	2.27E-01	1.04E-08	−1.41E-01	1.77E-05	3.19E-01	1.96E-10	−2.05E-01	3.60E-03
cg13852730	−6.23E-02	6.69E-03	2.21E-02	4.32E-01	−6.17E-02	6.30E-03	−4.80E-02	1.83E-01	1.42E-01	3.75E-03
cg17971328	−1.28E-01	5.30E-04	6.81E-02	1.36E-01	−1.28E-01	4.57E-04	−4.80E-02	4.13E-01	2.27E-01	4.00E-03
cg03915940	−4.48E-02	6.38E-02	−1.03E-02	7.26E-01	−4.43E-02	6.20E-02	−8.22E-02	2.89E-02	1.47E-01	4.10E-03
cg27182159	−9.43E-02	9.53E-03	8.37E-02	5.76E-02	−9.29E-02	9.16E-03	−2.12E-02	7.04E-01	2.19E-01	4.29E-03
cg14614754	−1.69E-01	1.19E-04	−1.14E-01	3.00E-02	−1.70E-01	8.79E-05	−2.39E-01	3.64E-04	2.62E-01	4.41E-03
cg16775460	−4.81E-02	3.39E-02	−3.27E-02	2.46E-01	−4.88E-02	2.89E-02	−1.03E-01	4.60E-03	1.40E-01	4.47E-03
cg14170437	−3.56E-02	3.88E-03	5.60E-02	1.28E-04	−3.62E-02	2.69E-03	8.96E-02	1.71E-06	−7.24E-02	5.21E-03
cg27097575	−5.02E-02	1.10E-02	2.11E-02	3.75E-01	−4.94E-02	1.08E-02	−3.47E-02	2.53E-01	1.16E-01	5.28E-03
cg25155298	−9.70E-02	1.84E-03	1.93E-02	6.14E-01	−9.68E-02	1.59E-03	−7.45E-02	1.31E-01	1.85E-01	5.35E-03
cg25747655	−7.41E-02	1.43E-05	−3.57E-02	8.05E-02	−7.39E-02	1.06E-05	−8.25E-02	1.62E-03	9.78E-02	6.44E-03
cg24379915	−1.25E-01	2.56E-04	7.48E-02	7.44E-02	−1.24E-01	2.40E-04	−2.44E-02	6.52E-01	1.95E-01	7.56E-03
cg12992443	−8.05E-02	1.63E-04	−3.14E-02	2.27E-01	−8.06E-02	1.27E-04	−9.12E-02	6.88E-03	1.20E-01	8.54E-03
cg21466736	−1.10E-01	3.77E-05	−3.31E-02	3.08E-01	−1.11E-01	2.89E-05	−1.07E-01	1.05E-02	1.48E-01	8.88E-03
cg20778199	−1.59E-01	2.28E-05	3.99E-02	3.84E-01	−1.58E-01	1.88E-05	−6.30E-02	2.83E-01	2.09E-01	8.97E-03
cg04729173	−3.40E-02	1.77E-01	2.43E-02	4.31E-01	−3.35E-02	1.77E-01	−4.58E-02	2.51E-01	1.40E-01	9.34E-03
cg03482600	−9.88E-02	1.51E-03	−1.09E-02	7.77E-01	−9.97E-02	1.20E-03	−9.99E-02	4.58E-02	1.74E-01	9.63E-03
cg18504937	−1.11E-01	4.99E-05	−1.98E-02	5.44E-01	−1.10E-01	4.24E-05	−9.01E-02	3.06E-02	1.48E-01	9.78E-03
cg16098304	−6.52E-02	1.29E-03	1.03E-02	6.77E-01	−6.49E-02	1.16E-03	−4.45E-02	1.63E-01	1.11E-01	1.03E-02
cg12989650	−6.79E-02	1.01E-02	−1.85E-02	5.67E-01	−6.81E-02	9.12E-03	−8.91E-02	3.13E-02	1.44E-01	1.07E-02
cg25766801	−4.00E-02	3.57E-02	8.73E-03	7.06E-01	−3.98E-02	3.40E-02	−4.29E-02	1.54E-01	1.04E-01	1.09E-02
cg00095276	−1.20E-01	1.40E-03	2.88E-02	5.24E-01	−1.18E-01	1.45E-03	−6.65E-02	2.44E-01	1.99E-01	1.12E-02
cg06381350	−8.56E-02	7.07E-03	7.21E-02	6.59E-02	−8.47E-02	6.99E-03	−1.50E-02	7.68E-01	1.72E-01	1.19E-02
cg03467813	−9.25E-02	1.25E-02	9.82E-02	2.89E-02	−9.14E-02	1.23E-02	3.48E-03	9.52E-01	1.96E-01	1.28E-02
cg05435065	−7.63E-02	2.10E-04	1.88E-01	1.19E-14	−7.32E-02	3.30E-04	2.35E-01	4.74E-15	−1.06E-01	1.34E-02
cg07387813	−1.17E-01	3.22E-05	1.89E-01	1.91E-08	−1.14E-01	4.33E-05	2.55E-01	1.05E-09	−1.47E-01	1.35E-02
cg10964388	−1.32E-01	4.43E-05	1.70E-02	6.62E-01	−1.31E-01	4.29E-05	−6.47E-02	1.93E-01	1.67E-01	1.38E-02
cg02776750	−7.97E-02	2.60E-03	1.70E-02	6.02E-01	−7.96E-02	2.38E-03	−5.61E-02	1.92E-01	1.41E-01	1.42E-02
cg17391741	−7.34E-02	3.48E-04	−3.07E-02	2.15E-01	−7.32E-02	3.05E-04	−8.35E-02	9.28E-03	1.07E-01	1.42E-02
cg24790419	−8.02E-02	7.48E-05	−1.29E-02	5.83E-01	−8.11E-02	4.47E-05	3.40E-02	2.62E-01	−1.03E-01	1.45E-02
cg07598331	−6.86E-02	4.49E-03	6.15E-02	3.95E-02	−6.79E-02	4.41E-03	−3.64E-03	9.26E-01	1.28E-01	1.50E-02
cg19017254	−4.71E-02	4.84E-02	1.11E-02	7.04E-01	−4.65E-02	4.90E-02	−5.01E-02	1.83E-01	1.24E-01	1.54E-02
cg12623107	−1.81E-01	4.18E-07	−1.13E-01	7.40E-03	−1.81E-01	2.98E-07	−1.97E-01	2.08E-04	1.77E-01	1.59E-02
cg01097768	−1.50E-01	4.66E-05	8.54E-02	5.49E-02	−1.49E-01	4.15E-05	−4.38E-03	9.39E-01	1.89E-01	1.62E-02
cg20806296	−1.11E-01	3.58E-03	−1.25E-02	7.85E-01	−1.10E-01	3.43E-03	−1.04E-01	7.52E-02	1.94E-01	1.64E-02
cg01111842	−1.25E-01	1.89E-07	1.87E-01	7.52E-11	−1.22E-01	3.10E-07	2.39E-01	1.02E-11	−1.21E-01	1.66E-02
cg09180820	−5.77E-02	1.21E-03	−4.20E-02	5.17E-02	−5.76E-02	1.08E-03	−8.63E-02	2.05E-03	9.08E-02	1.72E-02
cg02657160	−2.38E-01	9.70E-34	−4.78E-02	4.35E-02	−2.39E-01	1.15E-34	−9.89E-02	1.60E-03	9.94E-02	1.74E-02
cg13532410	−1.12E-01	5.23E-04	7.69E-02	4.95E-02	−1.11E-01	4.97E-04	−3.20E-03	9.50E-01	1.63E-01	1.77E-02
cg22530977	−2.88E-02	1.12E-01	5.46E-02	1.34E-02	−2.81E-02	1.16E-01	9.25E-03	7.47E-01	9.23E-02	1.78E-02
cg16226300	−1.60E-01	2.46E-08	1.59E-01	1.33E-06	−1.62E-01	8.53E-09	1.01E-01	1.37E-02	1.39E-01	1.82E-02
cg14791530	−1.31E-01	1.82E-06	1.06E-03	9.75E-01	−1.31E-01	1.44E-06	−6.94E-02	1.15E-01	1.39E-01	1.90E-02
cg18118795	−1.18E-01	2.53E-04	5.22E-02	1.75E-01	−1.17E-01	2.33E-04	−2.26E-02	6.46E-01	1.60E-01	1.92E-02
cg03562528	−1.04E-01	3.22E-05	−4.05E-02	1.77E-01	−1.04E-01	2.48E-05	−1.02E-01	9.14E-03	1.24E-01	1.96E-02
cg00887547	−7.10E-02	3.39E-04	2.04E-01	2.53E-18	−6.82E-02	5.15E-04	2.45E-01	1.58E-17	−9.54E-02	2.08E-02
cg19134728	−4.23E-02	6.10E-02	1.66E-02	5.52E-01	−4.21E-02	5.91E-02	−4.15E-02	2.58E-01	1.13E-01	2.10E-02
cg25428612	−4.77E-02	8.54E-03	8.14E-02	1.12E-04	−4.92E-02	5.91E-03	1.20E-01	8.70E-06	−8.65E-02	2.23E-02
cg12279175	−5.73E-02	2.87E-02	7.08E-02	2.87E-02	−5.60E-02	3.11E-02	5.29E-03	9.01E-01	1.28E-01	2.45E-02
cg27074221	−8.33E-02	2.34E-02	1.03E-01	2.56E-02	−8.19E-02	2.48E-02	8.81E-03	8.85E-01	1.82E-01	2.45E-02
cg17750043	−3.83E-02	9.68E-02	7.64E-02	6.50E-03	−3.67E-02	1.09E-01	2.23E-02	5.41E-01	1.09E-01	2.72E-02
cg05331472	−8.52E-02	1.45E-03	3.63E-02	2.68E-01	−8.47E-02	1.40E-03	−2.71E-02	5.27E-01	1.26E-01	2.86E-02
cg23611710	−8.26E-02	2.18E-02	1.50E-01	1.23E-03	−8.03E-02	2.50E-02	5.49E-02	3.75E-01	1.76E-01	2.99E-02
cg23629792	−1.39E-01	7.01E-04	−5.83E-02	2.32E-01	−1.39E-01	6.57E-04	−1.46E-01	1.80E-02	1.84E-01	3.14E-02
cg25051052	−7.57E-02	2.85E-03	1.65E-01	1.21E-07	−7.37E-02	3.43E-03	1.05E-01	1.04E-02	1.18E-01	3.19E-02
cg01476047	−1.10E-01	1.50E-03	1.42E-01	9.38E-04	−1.07E-01	1.78E-03	5.92E-02	2.94E-01	1.61E-01	3.20E-02
cg18334819	−5.47E-02	3.54E-02	8.13E-02	1.13E-02	−5.37E-02	3.71E-02	1.92E-02	6.51E-01	1.21E-01	3.25E-02
cg07399636	−1.35E-01	7.45E-05	−6.39E-02	1.13E-01	−1.36E-01	5.58E-05	−1.36E-01	8.08E-03	1.51E-01	3.34E-02
cg24793014	−7.85E-02	2.85E-04	5.98E-02	2.64E-02	−7.78E-02	2.90E-04	9.34E-03	7.91E-01	1.01E-01	3.39E-02
cg14743683	−3.65E-02	3.55E-02	−2.75E-02	1.85E-01	−3.63E-02	3.49E-02	−6.40E-02	1.60E-02	7.79E-02	3.45E-02
cg09166536	−1.11E-01	4.87E-03	−5.05E-03	9.13E-01	−1.10E-01	5.09E-03	−8.51E-02	1.46E-01	1.72E-01	3.49E-02
cg27536870	−2.51E-02	1.40E-01	5.07E-02	1.32E-02	−2.43E-02	1.49E-01	1.39E-02	6.00E-01	7.58E-02	3.59E-02
cg14007706	−8.66E-02	3.82E-05	−8.93E-02	3.69E-04	−8.65E-02	3.26E-05	−1.33E-01	4.02E-05	9.29E-02	3.75E-02
cg21898708	−3.64E-02	7.50E-03	1.30E-01	1.09E-15	−3.62E-02	7.32E-03	1.57E-01	3.52E-14	−5.95E-02	3.90E-02
cg23879460	−6.06E-02	1.99E-03	2.16E-02	3.62E-01	−6.01E-02	1.95E-03	−2.08E-02	5.02E-01	8.63E-02	4.02E-02
cg05880945	−1.18E-01	1.50E-05	1.45E-01	8.56E-06	−1.18E-01	1.47E-05	1.98E-01	1.30E-06	−1.19E-01	4.06E-02
cg04607032	−1.73E-01	1.18E-06	−1.16E-01	5.17E-03	−1.74E-01	8.92E-07	−1.86E-01	3.94E-04	1.48E-01	4.18E-02
cg26000722	−1.46E-01	4.89E-08	1.75E-01	2.31E-08	−1.43E-01	7.01E-08	2.23E-01	6.67E-09	−1.13E-01	4.36E-02
cg23348743	−5.73E-02	5.94E-03	2.89E-02	2.47E-01	−5.66E-02	6.14E-03	−1.26E-02	6.94E-01	8.83E-02	4.56E-02
cg02882813	−8.91E-02	2.38E-04	1.29E-02	6.63E-01	−8.89E-02	2.19E-04	−4.06E-02	3.01E-01	1.05E-01	4.57E-02
cg03811905	−4.84E-02	6.74E-03	1.01E-02	6.41E-01	−4.82E-02	6.50E-03	−2.81E-02	3.25E-01	7.69E-02	4.62E-02
cg14606129	−1.37E-01	3.39E-04	−7.51E-02	9.34E-02	−1.37E-01	3.09E-04	−1.49E-01	9.26E-03	1.58E-01	4.63E-02
cg10583473	−4.00E-02	6.31E-02	3.20E-03	9.03E-01	−3.99E-02	6.18E-02	−4.28E-02	2.15E-01	9.17E-02	4.88E-02
cg18766608	−6.00E-02	1.17E-02	4.34E-02	1.31E-01	−5.92E-02	1.22E-02	−6.21E-03	8.68E-01	9.96E-02	4.93E-02

*Values bolded indicate loci significantly predicted by the interaction of smoking and mMTHFR after Bonferroni correction for 909 tests (p < 5.500-05)*.

As demonstrated in Table 4, among loci hyper methylated in response to smoking, 90.4% of interactions were negatively valenced, indicating a more positive slope for those with lower m*MTHFR*. Conversely, among loci hypo methylated in response to smoking, 93.6% were positive, indicating a more negative slope for those with lower m*MTHFR*. In each case, there were consistent, but different directions of effect in the interaction of m*MTHFR* with smoking for hyper vs. hypo methylating loci. The consistently different effects resulted in a highly significant chi-square (1) = 194.299, *p* = 3.664 × 10^−44^. Across both hypo and hyper methylated loci, those with lower m*MTHFR* were found to have an exaggerated response to smoking.

**Table 4:** Comparison of direction of effect of interactions between mMTHFR and smoking at hypo methylating (Non-smoker > Smoker) vs. hyper methylating loci (Smoker > non-smoker) showing consistency across loci and divergence for loci showing hypo vs. hyper methylation in response to smoking.

	**Sign of Interaction term**	
	**Positive**	**Negative**	**Total**
Smoker > non-smoker	7 (9.6%)	66 (90.4%)	73
Non-smoker > Smoker	205 (93.6%)	14 (6.4%)	219
Total	212	80	292

*Chi-square = 194.299, df = 1, p = 3.664e-44*.

**Figure 1:**
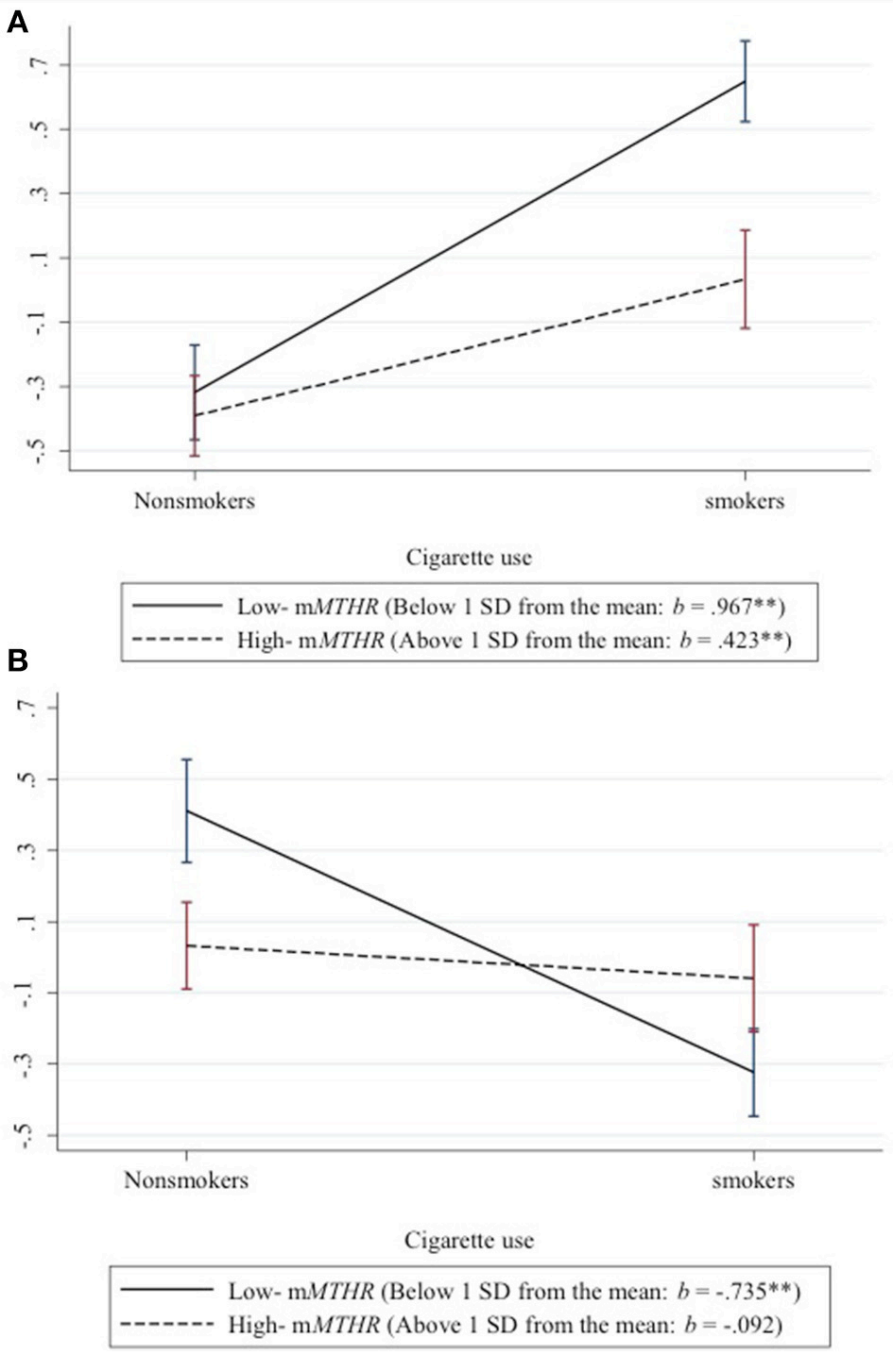
Differential response of hypo and hyper methylation in response to smoking status as a function of mMTHFR. Interactions are explicated by plotting the regression lines for values 1 sd above and below the mean on mMTHFR. **(A)** The average interaction for all loci showing a hyper methylating response to smoking indicates an exaggerated hyper methylating response among those with lower mMTHFR and a blunted hyper methylating response among those higher in mMTHFR. **(B)** The average interaction for loci showing a hypo methylating response to smoking indicates an exaggerated hypo methylating response among those with lower mMTHFR, but a blunted hypo methylating response among those higher in mMTHFR.

To better characterize the effect across loci, we standardized and then averaged across the 219 hypo methylating loci showing a positive interaction between smoking status and m*MTHFR* to create a hypo methylation index. Likewise we standardized and then averaged across the 73 hyper methylating loci showing a negative interaction between smoking and m*MTHFR* to create a hyper methylation index. We then graphed the resulting average moderated response to smoking status in Figure 1. Interactions are explicated by plotting the regression lines for values 1 sd above and below the mean on m*MTHFR*. As can be seen in panel A of Figure 1, the average interaction for loci showing a hyper methylating response to smoking is the result of an exaggerated hyper methylating response among those with lower m*MTHFR*, but a blunted effect among those higher in m*MTHFR*. Conversely, as shown in panel B of Figure 1, the average interaction for loci showing a hypo methylating response to smoking is the result of an exaggerated hypo methylating response among those with lower m*MTHFR*, but a blunted response among those higher in m*MTHFR*. This cross over interaction resulted in a lack of correlation between the hypo methylation index and m*MTHFR* across the whole sample (smokers and non-smokers), whereas the hyper methylation index was highly correlated with m*MTHFR* across the whole sample, as shown in Table 1.

Next, to control for potentially confounding effects, each methylation index was regressed against smoking, m*MTHFR*, and smoking^*^m*MTHFR*, controlling sex, age, diet, and mixed cell population effects. As demonstrated in Table 5, the interaction of smoking status and m*MTHFR* on the hyper methylating index comprising 73 loci is robust to the introduction of controls for sex, age, diet, and cell-type variation. Likewise the interaction of smoking status and m*MTHFR* on the hypo methylating index comprising 219 loci is robust to the introduction of controls.

**Table 5:** Linear regression models examining association of smoking status and mMTHFR and their interaction on indices of overall hypo and hyper methylation in response to smoking controlling for demographic variables, diet, and major cell type variation, indicating robust interaction effects.

	**Hypo methylated index (205 loci)**				**Hyper methylated index (66 loci)**			
**Model**	**Model 1A**		**Model 2A**		**Model 1B**		**Model 2B**	
**Parameters**	**b**	***p*-value**	**B**	***p*-value**	**b**	***p*-value**	**b**	***p*-value**
Smoking status	−0.424**	7.8e-09	−0.413**	2.8e-09	0.705**	4.5e-19	0.695**	8.0e-20
mMTHFR	−0.066	0.453	−0.410**	2.6e-04	−0.370**	4.3e-05	−0.080	0.476
Smoking status × mMTHFR			0.697**	4.2e-06			−0.589**	1.2e-04
Males	0.033	0.682	0.007	0.925	−0.135	0.100	−0.113	0.153
Age	−0.009*	0.021	−0.010**	0.006	0.001	0.724	0.002	0.545
Diet	0.028	0.410	0.028	0.384	−0.047	0.168	−0.047	0.152
CD8+T cells	−2.379**	1.3e-05	−2.376**	4.0e-06	−0.938†	0.078	−0.941†	0.066
CD4+T cells	0.264	0.503	−0.014	0.969	−0.817*	0.039	−0.582	0.129
Natural killer cells	−3.165**	2.6e-07	−3.006**	2.2e-07	1.322*	0.026	1.188*	0.038
B cells	−3.440**	7.5e-15	−3.051**	4.2e-13	1.409**	0.001	1.081**	0.007
Monocytes	1.279*	0.010	0.866†	0.067	−0.417	0.396	−0.068	0.887
Constant	1.433**	5.2e-06	1.576	1.6e-07	−0.137	0.651	−0.258	0.380
R-square	0.503	0.562	0.490	0.533

† *p ≤ 0.10*;

* *p ≤ 0.05*;

** *p ≤ 0.01 (two-tailed tests)*.

We then examined whether moderation of the CpG with the strongest evidence of moderation by *mMTHFR*, cg19859270, was also present after the introduction of controls. As shown in Table 6, the interaction of smoking status and m*MTHFR* on the effect of m*MTHFR* on smoking-induced hypomethylation of cg19859270 was robust to the introduction of controls for sex, age, diet, and cell-type variation. After the introduction of controls, the interaction effect is Beta = 0.073, *t* = 4.47, *p* = 0.000014. Results of the regression analysis indicated that levels of B cells and monocytes, calculated by methylomic indicators, were also significantly associated with cg19859270.

**Table 6:** Linear regression of smoking status and mMTHFR and their interaction on Cg19859270, an index of GPR15 proliferation, controlling for demographic variables, diet, and major cell type variation.

	**cg19859270**			
**Model**	**Model 1**		**Model 2**	
**Parameters**	**b**	***p*-value**	**b**	***p*-value**
Smoking status	−0.075**	3.8e−18	−0.074**	3.9e−19
mMTHFR	−0.037**	0.001	−0.073**	1.5e−08
Smoking status × mMTHFR			0.073**	1.4e−05
Males	0.008	0.400	0.005	0.571
Age	−0.001†	0.089	−0.001*	0.040
Diet	0.002	0.593	0.002	0.576
CD8+T cells	−0.033	0.569	−0.033	0.552
CD4+T cells	0.039	0.385	0.009	0.835
Natural killer cells	−0.045	0.488	−0.028	0.643
B cells	−0.346**	7.4e−13	−0.306**	4.0e−11
Monocytes	0.196**	0.001	0.153**	0.004
Constant	0.903**	1.1e−62	0.918**	2.4e−66
R-square	0.549	0.598

† *p ≤ 0.10*;

* *p ≤ 0.05*;

** *p ≤ 0.01 (two-tailed tests)*.

*N = 180*.

Because some loci have demonstrated dose-dependent methylation changes due to smoking (Shenker et al., 2013b; Ambatipudi et al., 2016; Joehanes et al., 2016; Marabita et al., 2017), we examined whether methylation change associated with self-reported consumption of cigarettes over time would demonstrate similar moderating effects by m*MTHFR* as those seen with binary smoking status. Using identical models as described above, the hyper and hypo methylated indices were regressed against smoking consumption, m*MTHFR*, their interaction term, and controls for sex, age, diet, and cell-type variation. As shown in Supplemental Table 1, the interaction terms for smoking consumption^*^m*MTHFR* remained significant predictors of both the hyper and hypo methylation indices when average daily consumption of cigarettes over the five waves and 11 years of the study, rather than presence or absence of smoking in the last 12 months, was included as a predictor. Similarly, for cg19859270, a model substituting self-reported level of smoking consumption for binary smoking status also showed a significant interaction effect for smoking^*^m*MTHFR*, as shown in Supplemental Table 2.

Lastly, we examined whether moderating effects of m*MTHFR* on indices of hyper and hypo methylated loci would be present under more stringent conditions, specifically including only those loci showing evidence of moderation by m*MTHFR* after Bonferroni correction. Therefore, we constructed a hyper methylated index comprising the 1 hyper methylated locus and a hypo methylated index comprising the 60 hypo methylated CpGs with significant interaction effects of smoking by m*MTHFR* even after Bonferroni correction. The Bonferroni corrected hypo and hyper methylated indices were regressed against smoking consumption, m*MTHFR*, the interaction of smoking with m*MTHFR*, along with controls for sex, age, diet, and cell-type variation, using statistical procedures identical to those described above for the full indices containing 66 hyper methylated and 205 hypo methylated loci. For both of the more stringent indices of hyper and hypo methylated loci, regression parameters for both smoking consumption, m*MTHFR*, and their interaction term remained significant, as indicated in Supplemental Table 3.

## Discussion

As an initial test of the hypothesized role of m*MTHFR* in moderating epigenetic responses to smoking, we examined its effects on changes in methylation at loci previously associated with smoking in a cohort of middle age African Americans. We examined three possible patterns to distinguish effects potentially attributable to (1) greater m*MTHFR* indicating lower folate availability and so preferentially amplifying hypo methylation responses, (2) greater m*MTHFR* indicating reduced AHR activity secondary to earlier and greater activation of the AHRR regulatory response and so reduced epigenetic remodeling induced by prolonged over activity of the xenobiotic pathway, or (3) greater m*MTHFR* indicating reduced AHR activity and so amplified epigenetic remodeling induced by less efficient removal of cigarette smoking related toxins.

We found a strongly consistent effect of moderation by m*MTHFR* of both the hypo methylating and hyper methylating effects of smoking at the 909 loci previously identified as being smoking associated in peripheral blood in African Americans (Dogan et al., 2014). Furthermore, this moderating effect was robust with regard to controls for sex, age, diet, and cell-type variation, all of which can impact peripheral methylation signatures. The direction of effect of moderation was consistent, with greater m*MTHFR* strongly associated with a blunted effect of smoking on methylation change at both hypo methylating and hyper methylating sites. The moderating effect of m*MTHFR* remained present when indices were restricted to only those hypo methylating and hyper methylating sites showing evidence of moderation by m*MTHFR* at a Bonferroni-corrected level of significance. Furthermore, there were no differences in patterns of effects observed between regression analyses using binary smoking status vs. those using self-reported level of cigarette consumption over time.

The pattern we observed is consistent with hypothesis 2, that activation of the xenobiotic pathway by AHR in smokers is a driver of genome wide changes in methylation, and that because this effect is reduced in high m*MTHFR* individuals, who exhibit a more robust regulatory response to AHR, they show less pronounced epigenetic remodeling in response to prolonged cigarette smoking. Conversely, our results did not support the hypotheses that greater regulation of AHR by AHRR would lead to exaggerated epigenetic signatures of smoking due to reduced clearance of toxins, or that greater m*MTHFR* would amplify only the hypo methylating effects of smoke through decreased folate availability.

Accordingly, we conclude that m*MTHFR* is important in understanding the long-term effects of smoking on remodeling of methylation and may help explain individual variability in the impact of smoking on disease risk. The mechanism underlying this plasticity requires additional examination, but plausibly involves the central role of MTHFR in modulating the regulatory response to AHR activation. However, the mechanisms underlying specific patterns of hypo and hyper methylation at smoking associated loci are likely diverse and are in need of further exploration. Factors including age, gender and diet may have discrepant effects on mechanisms underlying smoking associated change at specific loci. Our rough measure of nutritional status, as indexed by dietary habits, did not show any relationship with m*MTHFR*, though more nuanced measures of diet and measurement of specific nutrients such as folate could show an influence of methylation patterns.

The *MTHFR* 677C>T polymorphism has been reported to be associated with changes in methylation of *MTHFR* (Fernandes et al., 2018; Nash et al., 2018), and is an additional factor that could explain some of the differences in response to smoking observed in our sample. However, given the low frequency of the mutation in African Americans, with TT homozygotes estimate at 1–2% (Botto and Yang, 2000), the mutation is unlikely to be a significant source of variation in this sample. Indeed, in prior work with an ethnically similar sample, we found a low frequency and no significant main or buffering effect of *MTHFR* 677C>T polymorphism on the association of smoking and demethylation across *AHRR* among nascent smokers (Beach et al., 2017). Nevertheless, inability to completely control for the possible influence of the *MTHFR* genotype is a limitation of the current study.

Interestingly, although robustly related to smoking status and consumption, we did not observe exaggerated hypo methylation of cg05575921 among those higher on m*MTHFR*, as previously reported by Beach and colleagues (Beach et al., 2017) for nascent smokers. This may be due to the fact that that prior study examined two cohorts of young adult smokers as opposed to the older population examined in the current study. Specifically, it is possible that low m*MTHFR* individuals who have a blunted hypo methylating response to smoking at AHRR may nevertheless “catch up” to high m*MTHFR* individuals, who demonstrate a more rapid initial change, over time. In line with the current results, the initial rapid demethylation of cg05575921 among smokers with high m*MTHFR* would be coupled with slower demethylation in response to continued smoking, as it is for other hypomethylating loci. Thus, over time the difference in hypomethylation at cg05575921 between those with high vs. low m*MTHFR* would tend to disappear. Testing this developmental hypothesis will require longitudinal data with multiple assessments over time beginning with nascent smokers and continuing into middle age.

We found that the strongest moderating impact of m*MTHFR* on methylation change in response to smoking was at the CpG cg19859270, located in the gene *GPR15*. This locus was previously identified as the most strongly associated with smoking in another study of African Americans (Dogan et al., 2014), and the pattern of hypo methylation of cg19859270 has been shown by Bauer et al. (2015) to directly correlate with an increased population of GPR15+ T cells, which may help explain the pattern of cg19859270 hypomethylation observed in whole blood. Given that this pattern represents expansion of a specific cell population, it will be useful in future investigations to examine the role of methyl donor group availability in the observed effect, in addition to more general effects attributable to regulatory response to AHR activation. Indeed, others have shown that T-cell proliferation is inhibited by folate deficiency, suggesting a role for MTHFR in moderating the effect of smoking on expansion of this cell population (Courtemanche et al., 2004). At present, it is unclear to what extent other changes in peripheral immune cell composition underlie smoking associated methylation signatures, or the impact of m*MTHFR* on this process. For some loci, like cg05575921, changes in methylation appear to be robust to variation in cell mixture and tissue origin (Monick et al., 2012; Tsai et al., 2018), whereas for others, like cg19859270, may be linked to more specific changes in immune composition (Bauer et al., 2015, 2016). Interestingly, in our study, the hypo methylation index was more strongly associated with major cell type variation than the hyper methylation index, suggesting that hypo methylating loci moderated by m*MTHFR* are more likely to be linked with cell specific expression patterns in smokers than are hyper methylating loci.

The clinical significance of an increase in the proportion of GPR15+ cells, reported to be as much as 10-fold in some smokers (Bauer et al., 2015), is not fully understood. Jointly, GPR15 and its natural ligand GPR15L (Suply et al., 2017) traffic T cells to the gut (Kim et al., 2013) and, under inflamed conditions, to the skin (Ocón et al., 2017). In addition, GPR15 has been linked with ulcerative colitis (Nguyen et al., 2015), rheumatoid arthritis (Cartwright et al., 2014), psoriasis (Chen et al., 2018), and multiple sclerosis (Ammitzbøll et al., 2018). It is plausible that an increase in the population of GPR15+ T cells in smokers contributes to gut dysbiosis (Benjamin et al., 2011) and disruption of intestinal mucosal integrity, in turn promoting systemic inflammatory processes and disease progression (Budden et al., 2017; Gonçalves et al., 2018). Because GPR15 may be both a potential therapeutic target for inflammatory disease progression in smokers and a biomarker for smoking (Dogan et al., 2015), further investigation of this specific pathway and the moderating influence of m*MTHFR* on GPR15 in smokers is warranted.

More broadly, the results of this study may inform efforts to integrate epigenetic information into “precision medicine” by pointing toward mechanisms that underlie individual variation in epigenetic responses to environmental exposures and their relationship to the development of disease states. Although cigarette smoking has been identified as a paradigmatic environmental exposure with epigenetic effects (Ladd-Acosta, 2015), it is possible that the moderating effects of m*MTHFR* seen in this study will be generalizable to other exposures, for example alcohol (Philibert et al., 2018). Advances in understanding this and other moderating effects of exposures with epigenetic effects will help improve both diagnostics and elucidation of mechanisms underlying disease. In terms of immediate clinical translation, our findings may contribute to improved ability to predict smoking status based on measurement of methylomic signatures of smoking in conjunction with m*MTHFR*.

Limitations of the current study include its small size and lack of secondary methods to confirm the assessment of m*MTHFR*. In addition, although our sample of African Americans is likely to harbor low rates of functional mutations at MTHFR, it is not known if the effects we observed will generalize to other populations with higher rates of functional mutations at *MTHFR* (Sherry et al., 2001). Likewise, repeated measures taken over several decades will be needed to better inform understanding of patterns of change, whereas the current investigation must rely on concurrent assessments of smoking, m*MTHFR*, and smoking related hypo and hyper methylation. Nonetheless, the results provide an important starting point for predicting individual differences in response to smoking.

In brief, although the effect needs to be verified in additional samples and across a wider range of smoking associated loci, the impact of smoking on long-term changes in hyper and hypo methylation is moderated by m*MTHFR*. In both cases, those with lower levels of m*MTHFR* showed a greater effect of smoking on long-term remodeling of methylation, i.e., greater “methylation plasticity”. Of particular interest was a potentiation of hypo methylation of cg19859270, which signals the expansion of a particular T cell subpopulation expressing GPR15 with clinical disease relevance to smokers.

## Data Availability Statement

The full dataset of methylation data for 909 loci analyzed in this study are available in Supplemental Table 4.

## Ethics Statement

This study was carried out in accordance with the recommendations of the Institutional Review Boards of the University of Georgia and University of Iowa with written informed consent from all subjects. All subjects gave written informed consent in accordance with the Declaration of Helsinki. The protocol was approved by the Institutional Review Boards of the University of Georgia and University of Iowa.

## Author Contributions

RP and SB obtained funding and collected and characterized the subjects in the study. SB and M-KL conducted the analyses and wrote the initial draft of the manuscript. AA contributed to interpretation of findings and critically revised the manuscript.

### Conflict of Interest Statement

RP is the Chief Executive Officer of Behavioral Diagnostics and inventor on a number of granted and pending patent applications with respect to both alcohol and tobacco consumption related to the material discussed herein. The use of cg05575921 status to determine smoking status is protected by US Patents 8,637,652 and 9,273,358. SB has filed for a provisional patent to cover use of mMTHFR for quantification of smoking exposure and other potential applications. The remaining authors declare that the research was conducted in the absence of any commercial or financial relationships that could be construed as a potential conflict of interest.
